# Utility of Fasting C-Peptide for the Diagnostic Differentiation of Patients with Type 1, Type 2 Diabetes, MODY, and LADA

**DOI:** 10.3390/life14050550

**Published:** 2024-04-25

**Authors:** Ricardo Alemán-Contreras, Rita A. Gómez-Díaz, Maura E. Noyola-García, Rafael Mondragón-González, Niels Wacher, Aldo Ferreira-Hermosillo

**Affiliations:** 1 Servicio de Medicina Interna, Hospital de Especialidades, Centro Médico Nacional Siglo XXI, Instituto Mexicano del Seguro Social, Mexico City 06720, Mexico; ricardo_red@hotmail.com (R.A.-C.); mnoyola.g@gmail.com (M.E.N.-G.); 2 Unidad de Investigación Médica en Epidemiología Clínica, Hospital de Especialidades, Centro Médico Nacional Siglo XXI, Instituto Mexicano del Seguro Social, Mexico City 06720, Mexico; ritagomezdiaz@yahoo.com.mx (R.A.G.-D.); rafmg@hotmail.com (R.M.-G.); wachersniels@gmail.com (N.W.); 3 Unidad de Investigación Médica en Enfermedades Endocrinas, Hospital de Especialidades, Centro Médico Nacional Siglo XXI, Instituto Mexicano del Seguro Social, Mexico City 06720, Mexico

**Keywords:** autoimmunity, C-peptide, MODY, type 1 diabetes, type 2 diabetes

## Abstract

Background: The prevalence of obesity has increased in patients with type 1 diabetes (T1D) and latent autoimmune diabetes of the adult (LADA), limiting the use of clinical features such as the body mass index for its differentiation with type 2 diabetes (T2D). Additionally, some patients with maturity-onset diabetes of the young (MODY) or LADA are misdiagnosed as having T2D. The evaluation of autoantibodies and genetic testing are not fully available. We aimed to evaluate the utility of a widely available and less expensive diagnostic tool such as C-peptide to differentiate between T1D, T2D, MODY, and LADA. Methods: Our study included 38 patients with T1D, 49 with T2D, 13 with MODY, and 61 with LADA. We recorded anthropometric measurements, biochemical profiles, and antidiabetic treatment and determined C-peptide, anti-GAD65, and anti-IA2 antibodies. Results: C-peptide concentration differed significantly among populations (T1D: 0.2 ng/mL; T2D: 2.4 ng/mL; MODY: 1.14 ng/mL; LADA: 1.87 ng/mL). Through a ROC curve, we observed that the C-peptide cut-off point of 0.95 ng/mL allows differentiation between T1D and T2D (sensitivity 82%, specificity 77%); 0.82 ng/mL between T1D and LADA (sensitivity 82%, specificity 77%); and 1.65 ng/mL between T2D and MODY (sensitivity 72%, specificity 72%). Conclusions: C-peptide is useful for the diagnostic differentiation of patients with type 1, type 2 diabetes, MODY, and LADA.

## 1. Introduction

According to the Encuesta Nacional de Salud y Nutrición 2022 (National Health and Nutrition Survey 2022), the prevalence of people diagnosed with diabetes mellitus > 20 years of age in Mexico is 12.6% [1]. However, the prevalence of type 1 diabetes (T1D) and other types of diabetes, such as latent autoimmune diabetes of the adult (LADA), which is characterized by late development of autoimmunity [2], or Maturity Onset Diabetes of the Young (MODY) [3], in Mexico is unknown.

In the clinical setting, age, and body mass index (BMI) are widely used to guide the diagnosis of the type of diabetes a patient has, but they do not detect autoimmunity [4]. This leads to misdiagnosis and the use of inadequate treatments, such as oral antidiabetics in a patient with T1D or high doses of insulin instead of the administration of oral antidiabetics in some patients with T2D or MODY. This difficulty in diagnosis is increased in the case of youths with overweight/obesity [5]. Because of this, some studies have been proposed to differentiate between patients with and without autoimmunity, such as the determination of autoantibodies or the search for risk haplotypes in the human leukocyte antigen (HLA) complex, analogous to the major histocompatibility complex (MHC) [6]. However, these studies are expensive, and their availability is limited. 

Due to the above, other proposals have arisen for diagnostic differentiation, such as the determination of C-peptide concentrations [7]. The presence of C-peptide is associated with the degree of pancreatic ß-cell reserve, so a patient with T1D has undetectable concentrations after a period of 10 years, while the pediatric population recently diagnosed or patients with T2D or MODY persist with measurable secretion of this peptide [8]. Furthermore, patients with LADA show a lower concentration in comparison with T2D [9]. 

The objective of this study was to assess the utility of C-peptide for diagnostic differentiation between T1D, T2D, MODY, and LADA in the adult Mexican population and to evaluate its correlation with clinical parameters.

## 2. Materials and Methods

A cross-sectional analytical study was performed on consecutive patients previously diagnosed with T1D, T2D, LADA, or MODY attending the Diabetes Clinic at the Hospital de Especialidades of Centro Médico Nacional Siglo XXI, a tertiary referral center. This study was approved by the Local Research and Ethics in Health Research Committee (F-2016-3601-3). We included patients that attended at least three visits in the last year, aged 18–70 years, with previous clinical diagnosis of T1D, T2D, or MODY, classified according to the standards of the American Diabetes Association (ADA) [10], or with LADA, according to the criteria of the Immunology of Diabetes Society [2]. Patients with T1D were diagnosed during childhood with the presence of an islet autoantibody and an immediate requirement for insulin (in comparison with patients with LADA who could be treated with oral antidiabetic drugs for at least 6 months). We included patients clinically diagnosed with HNF1A-MODY. Those patients had an increase in serum glucose > 90 mg/dL in the oral glucose tolerance test, the presence of glycosuria when serum glucose was lower than 180 mg/dL in several follow-up tests, a normal concentration of HDL-cholesterol (HDL-c), and an initial sensitivity to sulfonylurea drugs with insulin requirement after a year. Furthermore, those patients were assessed through the MODY probability calculator available at www.diabetesgenes.org (accessed on 1 February 2024) [11], that is based on the prediction model performed by Shields et al. that detects the chance of having MODY when the probability cutoff is >25% [12]. For improving the detection capacity of the calculator, we applied the corrected cutoff proposed by Silva Santos et al. of >36% [13]. This cutoff increases the predictive positive value by 74.4% and the predictive negative value by 73.5% [13].

This study included all the patients recruited from their diabetes appointments who accepted participation. Their clinical diagnosis was verified through their medical records. We excluded patients with other forms of diabetes. The objective of this study was explained to all participants, who gave their written informed consent. The protocol was conducted in accordance with the Declaration of Helsinki. 

### 2.1. Anthropometric Measurements

During the office visit, age, sex, weight (kilograms), height (meters), and waist circumference (centimeters) were recorded. Only one of the investigators performed the anthropometric measurements using a calibrated scale with a stadiometer and flexible, non-expandable metric tape. The waist circumference (WC) was measured at the midpoint between the last rib and the upper border of the anterosuperior iliac spine. BMI was calculated as weight divided by the height squared, and the waist/height ratio (WHtR) was calculated.

### 2.2. Biochemical Determinations

After an 8 h fast, a sample of 6 mL of blood was taken in a BD Vacutainer tube (BD Franklin Lakes, NJ, USA) and centrifuged at 3150× *g* for 15 min to obtain serum. Glucose, total cholesterol (TC), and triglycerides were determined using a commercial kit (COBAS, Roche Diagnostics, Indianapolis, IN, USA) through photocolorimetry using a Roche Modular P800 spectrophotometer (Roche Diagnostics, Indianapolis, IN, USA). For the determination of HDL-c, an aliquot of serum was treated with polyethylenglicol and dextran sulfate and analyzed with a commercial kit for cholesterol (COBAS, Roche Diagnostics, Indianapolis, IN, USA). At the same time, 3 mL of blood was collected in a BD Vacutainer tube with EDTA-K3 (BD Franklin Lakes, NJ, USA) for evaluation of Glycated hemoglobin (HbA1c). HbA1c was evaluated through turbidometric immunoanalysis (COBAS, Roche Diagnostics, Indianapolis, IN, USA). LDL-cholesterol (LDL-c) was calculated through the Friedewald formula: LDL-c (mg/dL) = TC mg/dL − (HDL-c mg/dL + triglycerides mg/dL/5), whenever triglycerides were less than 400 mg/dL.

### 2.3. Determination of C-Peptides and Antibodies 

Determination of C-peptide was performed with a COBAS commercial kit (Roche Diagnostics, USA) in fresh serum (after an 8 h fast). The limit of detection of the test is 0.010–40 ng/mL, with a coefficient of inter-assay variation of 4.6% and intra-assay variation of 5%. All values below the limits of detection were considered the minimum. The test cross-reactivity with human insulin is 0.005%. Determination of antibodies anti-glutamate descarboxylase 65 (anti-GAD65) and anti-tyrosine phosphatase related to islet 2 antigen (anti-IA2) was performed through ELISA technique with a commercial kit, Medizym Brand (Medipan GMBH, Blankenfelde-Mahlow, Germany). For anti-GAD65, the cut-off for positivity is >5 IU/mL (specificity 98.6%, sensitivity 92.3%). The test analytical sensitivity reported is 0.8 IU/mL and functional 4 IU/mL, with coefficients of inter-assay variation of 7% and intra-assay variation of 4%. For anti-IA2, the cut-off for positivity is >10 IU/mL (specificity 98%, sensitivity 75%). The test analytical sensitivity reported is 0.5 IU/mL and functional sensitivity is 0.8 IU/mL, with coefficients of inter-assay variation of 6% and intra-assay variation of 5%. 

### 2.4. Statistical Analysis

We evaluated normality using a Kolmogorov–Smirnov test. Quantitative variables are described using medians with interquartile ranges due to the distribution of data. Qualitative variables are described using frequency and percentages. The association between qualitative variables was analyzed using the chi-squared test and an ANOVA if there were more than two groups. Receiver operating characteristic (ROC) curves were made of concentrations of C-peptide to identify the cut-off with optimum sensitivity and specificity that would allow diagnostic differentiation, with 95% confidence intervals. We also determined the area under the curve (AUC). The correlation between variables was analyzed using the Spearman test. We evaluated all the clinical and biochemical factors registered that could influence C-peptide concentration with a multiple regression analysis. A *p* < 0.05 was considered statistically significant. For statistical analysis, statistical packages SPSS version 20 and STATA version 11 were used. 

## 3. Results

The study included 161 patients: 22.3% initially diagnosed with T1D, 30.4% with T2D, 9.3% with MODY, and 37.9% with LADA; 59% of the patients were women, with a median age of 42 years (28–62 years). All patients have had more than 10 years since the diagnosis of diabetes. Patients with T2D were older, followed by patients with LADA, MODY, and finally patients with T1D. It was found that WC was greater in patients with T2D compared with T1D, MODY, or LADA, while WHtR was different between the various populations. BMI showed significant differences between patients with T1D and T2D, and LADA and T2D.

When comparing T1D and MODY, differences were observed in the glucose (*p* = 0.028) and C-peptide (*p* < 0.001) concentrations. When comparing MODY and LADA, only age (*p* = 0.007) was different (Table 1). Fasting glucose concentrations in patients with MODY (185 mg/dL) or LADA (190 mg/dL) were higher in comparison with those of patients with T1D (138 mg/dL) or T2D (135 mg/dL), without differences between T1D and T2D. C-peptide concentrations were significantly different between the different populations (T2D vs. T1D *p* < 0.001; T2D vs. MODY *p* = 0.028; T1D vs. LADA *p* < 0.001; T2D vs. LADA *p* = 0.019). There was no difference in C-peptide concentration between patients with T1D and MODY and between MODY and LADA. In order to evaluate if hyperglycemia influenced C-peptide levels, we excluded subjects with glucose levels higher than 11.1 mmol/L: 2 patients with T2D, 7 patients with T1D, 5 patients with MODY, and 27 patients with LADA. Even after those patients were excluded, C-peptide concentrations were significantly different among the groups (T2D vs. T1D *p* < 0.001; T2D vs. MODY *p* = 0.036; T1D vs. LADA *p* < 0.001; T2D vs. LADA *p* = 0.010) (Table 2).

The concentration of HbA1c, total cholesterol, triglycerides, LDL-c, HDL-c, and proteins in 24 h urine showed no difference between kinds of diabetes, with the exception of LDL-c in cases with LADA. The determination of anti-GAD 65 autoantibodies was performed in the whole population: 55% of the patients with T1D had positive anti-GAD65 antibodies, compared with 4% (2/49) of the patients with T2D. Both patients were classified as T2D due to their adequate glycemic control with oral antidiabetic drugs (HbA1c < 7%, 53 mmol/mol) despite disease duration. As for MODY, two patients presented positive antibodies; however, in accordance with their clinical and biochemical characteristics, they were re-classified as patients with T1D. Determination of anti-IA2 autoantibodies was carried out in all patients, with 42% of the population with T1D and 62% of LADA patients being positive. (Table 2).

Table 3 shows the antidiabetic treatment for the study groups. More than half of the patients in each group were under treatment with insulin, in response to the lack of metabolic control noted (see Table 2). It is remarkable that patients with MODY received more oral antidiabetic treatment in addition to insulin (77% received sulfonylurea and/or 54% metformin, with 69% receiving insulin). There were no differences in C-peptide levels between patients with (n = 31) or without (n = 130) sulfonylurea treatment (1.35 ng/mL [0.75–2.23] vs. 1.71 ng/mL [0.67–2.71], respectively; *p* = 0.764).

**Figure 1:**
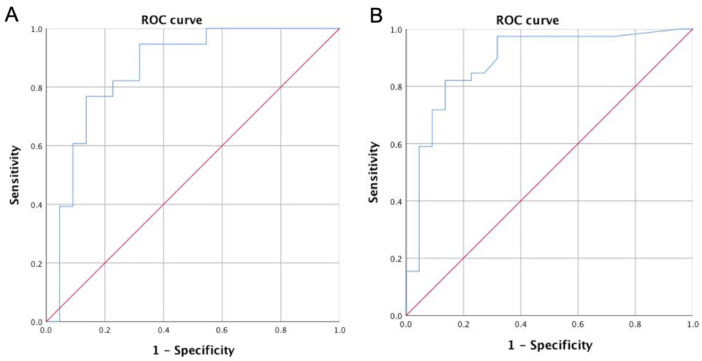
ROC curve for fasting C-peptide: (**A**) for diagnosis between type 1 and type 2 diabetes; (**B**) for diagnosis between type 1 diabetes and LADA.

The cut-off for fasting C-peptide was 0.95 ng/mL for diagnostic differentiation between T1D and T2D (sensitivity 82%, specificity 77%, AUC 0.88; Figure 1A); the cut-off for fasting C-peptide was 0.82 ng/mL for diagnostic differentiation between T1D and LADA (sensitivity 82%, specificity 77%, AUC 0.86; Figure 1B); and the cut-off for fasting C-peptide was 1.65 ng/mL for diagnostic differentiation between T2D and MODY (sensitivity 72%, specificity 72%, AUC 0.75).

It was observed that anti-GAD65 autoantibodies for the diagnosis of T1D have a sensitivity of 37%, specificity of 91%, positive predictive power (PPP) of 71%, negative predictive power (NPP) of 72%, false positives of 8%, and diagnostic certainty of 79%. Likewise, anti-IA2 autoantibodies have a sensitivity of 19%, specificity of 100%, PPP of 100%, NPP of 69%, and certainty of 87% for the diagnosis of T1D.

As shown in Table 4, a moderate correlation was observed between C-peptide and WC in the total study population and in patients with T2D. Likewise, in this group, a positive association was found with triglycerides and a negative correlation with HDL-c. In addition, a correlation was observed between C-peptide and weight in the total study population, as well as in patients with T1D and T2D. 

A multiple regression analysis was performed (Table 5), where 33% of C-peptide concentration was influenced by WC (ß = 0.468), insulin use (ß = −0.280), and sex (ß = 0.217). Upon dividing by groups, in T2D, 39% of C-peptide concentration was influenced by WC (ß = 0.496), sex (ß = 0.307), and insulin use (ß = −0.288), while in T1D, 19% of C-peptide concentration was influenced by BMI (ß = 0.482). In LADA, HbA1c (ß = −0.280) influenced 6% of C-peptide concentration. In MODY, C-peptide was not influenced by any studied variable. Neither age, diabetes duration, oral treatment used, nor creatinine clearance influenced C-peptide concentrations. Evaluating alternative regression models, we also corroborate that none of the other variables influenced C-peptide concentration.

## 4. Discussion

Type 1 diabetes was once considered the only form of diabetes in children and adolescents; however, there is a growing prevalence of T2D in that population due to increased overweight and obesity [14]. This situation has generated difficulty in the adequate classification of a patient diagnosed with diabetes, where one may face a patient with obesity, which generates insulin resistance (which orients us towards a diagnosis of T2D) [5] or a patient with autoimmunity that develops obesity due to over-insulinization and an inadequate diet (which directs us towards a patient with T1D with metabolic syndrome, also known as “double diabetes”) [15]. On the other hand, there are also patients without a history of insulin resistance that debut with insulin treatment-resistant hyperglycemia at different ages (which can orient us towards a diagnosis of MODY) [16] and patients that display a milder autoimmune process, slower ß-cell failure, and insulin independence for 6 to 12 months after diagnosis (which orient us towards a diagnosis of LADA) [17]. This entity is not rare, since it is estimated that almost 10% of patients diagnosed with T2D have LADA [18]. However, its clinical differentiation has become difficult due to overweight and adiposity, which are also risk factors for LADA [18].

One way to differentiate between patients with and without autoimmunity is the determination of autoantibodies [19]. Nevertheless, the detection of a single autoantibody is insufficient [20], and there is a possibility of not detecting them at the blood level (for example, in a patient with idiopathic T1D) [6]. Additionally, as observed in our study, the prevalence of antiGAD65 autoantibodies has been reported to be 4.3% [21] in patients with T2D. This might be related to the frequency of those autoantibodies even in the non-diabetic population (0.7–4.8%) [22].

Considering those difficulties, there is a need to use other biochemical resources that allow the adequate differentiation of patients with T1D, T2D, MODY, and LADA. C-peptide has been a useful tool in the adult population since patients with T1D show deficient levels of C-peptide 2 or 3 years after diagnosis, while patients with T2D or MODY persist with detectable levels [23]. In addition, there is the advantage of broad commercial availability, comparatively low cost, and easy availability [24]. For its determination, C-peptide requires immediate lab analysis due to its quick degradation [25]. Those conditions were considered for C-peptide quantification in our protocol. Regarding the different assays for its determination, C-peptide could also be quantified after 8 to 10 h of fasting or after stimulation with glucagon, intravenous/oral glucose, tolbutamide, sulfonylurea, or a mixed meal [26], but these techniques are not effective in the presence of insulin treatment. Despite the fact that fasting C-peptide has been criticized due to its limited ability to detect subtle levels, other studies have proven that it correlates with urinary C-peptide [26]. Urinary C-peptide levels had a high sensitivity for differentiating MODY from T1D or T2D [27], but had the disadvantage of being useful only in patients with adequate renal function. 

In our study, significant differences were presented between the C-peptide concentrations of patients with T1D, T2D, MODY, and LADA. Those differences were significant even after long-term disease duration and despite treatment. In fact, as demonstrated by Bouche et al., prolonged treatment with insulin increases the C-peptide response [28]. As mentioned, the majority of C-peptide is metabolized by the kidneys, with 5–10% excreted unchanged in the urine. In patients with chronic kidney disease, its precise quantification is difficult [25]. In our study, despite some differences in creatinine clearance among groups, all patients had a creatinine clearance higher than 60 mL/min/1.73 m^2^. Furthermore, creatinine clearance did not influence C-peptide concentrations in the multiple regression analysis.

C-peptide concentration declines over time in all types of diabetes [7]. It has been observed that the prevalence of detectable C-peptide varied from 19% in people diagnosed before the age of 15 with diabetes duration greater than 15 years to 92% in those with onset after 35 years and diabetes duration less than 5 years [29]. In China, patients with LADA had a rapid decline in C-peptide concentrations during the first 5 years of diagnosis, depending on the GAD autoantibody titration [30]. In T2D, there is a hyperinsulinemic phase due to insulin resistance that develops before the decline of ß-cells. During the prediabetes stage, a high proinsulin/C-peptide ratio is observed. After diabetes onset, C-peptide declines; however, it persists detectably for more than 20 years despite the use of insulin [29].

In Korea, in 223 patients with diabetes, levels of C-peptide 0.6 ng/mL excluded a diagnosis of T2D, while C-peptide levels > 3.0 ng/mL made a diagnosis of T1D improbable [31]. Likewise, Katz et al., in 175 patients, identified fasting concentrations of C-peptide of 0.85 ng/mL (83% sensitivity, 89% specificity) to distinguish the pediatric population with T1D from the population with T2D [32]. In our study, the cut-off of 0.95 ng/mL achieved a similar sensitivity (82%), while specificity was lower (77%). Genes et al. evaluated 104 patients with diabetes aged older than 16 years: 24 with HNF4A-MODY, 40 with T1D, and 40 with T2D; their mean age was 32 years, with fasting glucose levels > 200 mg/dL and HbA1c > 10%. In this group, C-peptide levels were different between T1D and T2D (0.86 [0.01–4.61] ng/mL vs. 2.38 [1.05–11.8] ng/mL, *p* < 0.001) and between T1D and MODY (with a C-peptide concentration of 1.78 [0.47–5.05] ng/mL, *p* = 0.003) [33]. Although they did not perform ROC curves, it is noteworthy that their C-peptide levels are similar to our proposed cut-off points. In another cohort study carried out in Sweden that included 2734 children recently diagnosed with diabetes, patients classified as T2D had the highest C-peptide concentration (5.5 ng/mL), followed by patients with MODY (3 ng/mL) and T1D (0.84 ng/mL) [8]. Despite that our population is quite different from our adult cohort, with more than 10 years since the diagnosis of diabetes, it supports that lower C-peptide levels are useful for detecting T1D. 

Fasting C-peptide levels in patients with LADA are lower when compared with those with T2D. However, ß-cell reserves differ among patients with LADA due to heterogeneity in the severity of the autoimmune process. This has been considered by an international expert panel that recommends a personalized treatment depending on three categories of random C-peptide: if levels are less than 0.90 ng/mL (0.3 nmol/L), a multiple-insulin regimen is suggested; those with levels between 0.90 and 2.0 ng/mL (0.3–0.7 nmol/L) could be treated with insulin in combination with other therapies in a flexible scheme; and those with levels higher than 2.0 ng/mL (0.7 nmol/L) could be treated according to T2D guidelines. In this group, they recommend repeating the C-peptide measurement if glycemic control deteriorates [34]. In our study, the mean C-peptide concentration in patients with LADA was 1.87 ng/mL, which suggests that they could benefit from a combined treatment with insulin and oral anti-diabetic drugs. Most importantly, we defined a C-peptide cut-off point of 0.82 ng/mL, which allows us to differentiate between T1D and LADA. Future studies could explore if this cut-off point could differentiate between those with autoimmune diabetes in longitudinal studies with a larger sample size that includes other ethnicities. 

Thunander et al. evaluated the utility of C-peptides to classify diabetes. They found that C-peptide was a better discriminator in comparison with age and BMI to identify those patients with at least one positive autoantibody (anti-GAD and/or anti-IA2). In fact, upon creating ROC curves (taking as an evaluation parameter the adequate classification of the patient), they observed that the highest values in the AUC were obtained by C-peptide, followed by age and BMI (AUC of 0.78, 0.68, and 0.66, respectively) [24]. In our study, the ROC curve for C-peptide had a greater AUC (0.88), which translates to greater discriminatory ability in comparison with that previously reported. However, BMI and age did not have adequate discriminatory ability. The ADA and the European Association for the Study of Diabetes (EASD) consensus report on the management of T1D in adults recommend evaluating C-peptide after 3 years since diagnosis in autoantibody-negative patients. They proposed that a cut-off point of 0.60 ng/mL (0.20 nmol/L) suggests a diagnosis of T1D, while values higher than 1.80 ng/mL (0.60 nmol/L) indicate T2D [35]. According to our results, a higher cut-off point could be proposed to differentiate T1D from T2D (0.95 ng/mL), while the cut-off point of 1.65 ng/mL also allows the differentiation between T2D and MODY in the Mexican population. Being able to adequately differentiate the type of diabetes that a patient has allows us to provide an appropriate and more effective treatment. For example, mainly insulin in patients properly diagnosed as having T1D, sulfonylureas in patients with MODY, or a multi-treatment approach in patients with T2D.

C-peptide has been correlated with various components of metabolic syndrome. Haban et al., in a study of older patients with T2D, found significant correlations between C-peptide and triglycerides, HDL-c, and various relations with different kinds of atherogenic indices (total cholesterol /HDL-c and TG/HDL-c), as well as BMI and leptin concentrations [36]. In our study, we observed a moderate correlation with fasting glucose both in the general population and in patients with T2D, showing no significance in patients with T1D or MODY. C-peptide and BMI showed a slight correlation in T1D and T2D and a negative correlation with HDL-c in patients with T2D. Considering the above, serum determination of C-peptide might constitute an efficient tool in the prediction of cardiovascular diseases in patients with T2D, permitting primary measures of prevention and opportune treatment.

Regarding the determination of autoantibodies in patients with T1D, it has been reported that the prevalence of anti-GAD oscillates between 60 and 85%, while the determination of anti-IA2 varies between 70 and 90% of patients [37]. In our population, the prevalence of anti-GAD was 55% and of anti-IA2 was 42%. The prevalence of anti-IA2 has not been reported previously in our country. In T1D, there is usually a high percentage of positivity for anti-GAD at diagnosis, but anti-IA2 positivity rises with disease progression [38]. However, another study from the same trial found that levels of C-peptide dropped over time, at least during the first two years [39]. In contrast, in LADA, levels of both anti-GAD and anti-IA2 decrease between one and ten years of disease duration [40]. In the case of LADA, evidence suggests an inverse relationship between anti-GAD and C-peptide [41].

The diabetes autoantibody standardization program (DASP) has proposed different cut-off points, as well as standardization of the reference ranges for the diagnosis of T1D [42]. In the DASP 2000 workshop, both anti-GAD and anti-IA2 showed high sensitivity (80 and 58%) and specificity (90 and 100%, respectively) [43]. In contrast, in our study, lower sensitivity was observed with greater diagnostic specificity for anti-GAD and anti-IA2 antibodies. Zinc transporter 8 (ZnT8) autoantibodies have been recognized as one of the major anti-islet autoantibodies, present in 63% of patients with T1D and in 27% of patients with LADA. It has been observed that a more complete autoantibody panel could increase the accuracy of differentiation between autoimmune and non-autoimmune diabetes [44]. In our institution, we were not able to perform anti-ZnT8 autoantibodies. In fact, its use is not widespread in Mexico. Furthermore, it is necessary to stress that the determination of antibodies is costly and is only performed in some research units in our institution; therefore, we propose the determination of C-peptide as a less expensive diagnostic tool that is easy to perform and that can be used in primary care facilities.

In regard to limitations, one might consider the scant number of patients with MODY and that all of them were selected from a single center in a specific region of Mexico. Additionally, our clinic belongs to a tertiary referral center, where we attend a higher number of patients with T1D, MODY, and LADA and fewer patients with T2D than expected nationwide. This could limit the generalizability of the findings, as the population might not be fully representative of all Mexicans. We expect to establish further collaborations with different clinics around Mexico and Latin America that will allow us to confirm our results. Furthermore, the cross-sectional design of the study impedes our ability to evaluate how the C-peptide level changes over time in patients with T2D, LADA, and MODY. We plan to perform a longitudinal study to evaluate the utility of C-peptide for monitoring disease progression in these different types of diabetes, and if its concentrations are related to response to treatment. Another limitation was the impossibility of performing genetic studies on our entire population. However, McDonald et al. have suggested that the presence or absence of islet antibodies, especially anti-IA2, is a good indicator of MODY vs T1D and that the more expensive genetic testing should only be administered if other clinical characteristics so warrant it [45]. In addition, we obtained a power calculation higher than 90% when comparing C-peptide concentrations among groups. Another limitation of the study is the time since the diagnosis of all types of diabetes in the population (10 years). Previous studies in patients with T1D have reported residual insulin secretion at the time of diagnosis and within 1–2 years after diagnosis [46]. Likewise, a large cohort from the T1D Exchange Clinic Network reported that the odds of having detectable C-peptide were 7% lower for every year increase in diabetes duration. Nevertheless, they also observed that 35% of the participants with 10–19 years since diagnosis still had detectable residual C-peptide [46]. Haupt et al., in the KID Study, observed that among patients with type 2 diabetes, C-peptide decreased with disease duration of 15–20 years, but still was in the high normal range even after this time [47]. Chaillous et al. also observed that among patients with LADA with tight metabolic control, C-peptide concentrations decreased, irrespective of age, gender, BMI, antibody titers, HbA1c, or treatment modality [48]. At this point, we must consider that patients in our study had variable ranges of glycemic control. It is well known that high blood glucose levels stimulate insulin secretion. Despite this, some studies have observed that chronic hyperglycemia (assessed through HbA1c) does not influence C-peptide levels [49]. Furthermore, neither diabetes duration nor glycemic control were associated with C-peptide levels in the logistic regression analysis performed in our study. 

Finally, we must consider that this study was conducted on the Mexican population. More studies need to be conducted on populations of different ethnicities to corroborate our findings.

## 5. Conclusions

C-peptide could be useful for the diagnostic differentiation of patients with T1D, T2D, and LADA. According to our results, a cut-off point of 0.95 ng/mL could differentiate between T1D and T2D, while a cut-off of 0.82 ng/mL could differentiate between T1D and LADA. Despite the significance, the cut-off of 1.65 ng/mL observed between T2D and MODY remains with a 25% misclassification and requires further investigation. These results are only applicable to patients with long-standing diabetes.

## Figures and Tables

**Table 1:** Comparison of anthropometric and clinical variables between patients with type 1 diabetes, type 2 diabetes, LADA, and MODY.

	T1D (n = 38 *)	T2D (n = 49)	MODY (n = 13 *)	LADA (n = 61)	p **				
					T2D vs. T1D	T2D vs. MODY	T1D vs. LADA	T2D vs. LADA	LADA vs. MODY
Age (years)	27	60	37	50	<0.001	<0.001	<0.001	0.010	0.007
	(23–35)	(46–71)	(25–52)	(43–59)					
Disease duration (years)	17 (11.1–24)	13.6 (11–16.9)	19 (11.2–30.4)	12 (8.2–14.7)	NS				
Female (%)	66	53	54	61	NS				
BMI (kg/m^2^)	24.4	29	26.4	25.9	0.012	NS	NS	0.013	NS
	(22.2–28.5)	(24.9–31.7)	(24.7–27.8)	(24.0–28.4)					
WC (cm)	88	105	91	92	<0.001	0.012	0.012	<0.001	NS
	(79–94)	(92–114)	(77–101)	(87–99)					
Female	85	107	91	92	<0.001	NS	0.015	0.009	NS
	(77–94)	(90–113)	(82–112)	(87–97)					
Male	90	105	85	94	0.008	0.010	NS	0.002	NS
	(80–102)	(94–116)	(70–96)	(86–100)					
WHtR	0.54	0.64	0.54	0.59	<0.001	0.014	0.001	0.002	NS
	(0.49–0.59)	(0.58–0.71)	(0.46–0.60)	(0.56–0.62)					
Female	0.53	0.66	0.57	0.59	<0.001	NS	<0.001	0.008	NS
	(0.48–0.58)	(0.60–0.74)	(0.50–0.72)	(0.57–0.63)					
Male	0.55	0.62	0.48	0.57	NS	0.025	NS	0.019	NS
	(0.49–0.62)	(0.55–0.67)	(0.42–0.55)	(0.50–0.60)					

T1D: type 1 diabetes; T2D: type 2 diabetes; LADA: Latent Autoimmune Diabetes in Adults; MODY: Maturity Onset Diabetes of the Young; NS: not significant; BMI: body mass index; WC: waist circumference; WHtR: waist to height ratio. The results of quantitative variables are given in median with interquartile range. * Two patients with MODY were reclassified as T1D due to the positivity of anti-GAD. Results of quantitative variables are given in median with an interquartile range. ** There were no statistical differences among patients with T1D vs. MODY.

**Table 2:** Comparison of biochemical and immunological variables between patients with type 1 diabetes, type 2 diabetes, LADA, and MODY.

	T1D (n = 38 *)	T2D (n = 49)	MODY (n = 13 *)	LADA (n = 61)	p **				
					T2D vs. T1D	T2D vs. MODY	T1D vs. LADA	T2D vs. LADA	LADA vs. MODY
Glucose (mg/dL)	138	135	185	190	NS	0.04	0.001	0.001	NS
	(81–188)	(115–176)	(116–268)	(139–254)					
HbA1c (%)	8.8	8.25	9.8	9.4	NS				
	(7.6–10.1)	(6.7–10.2)	(7.8–12.1)	(7.2–11.0)					
HbA1c (mmol/mol)	73	67	84	79	NS				
	(60–87)	(50–88)	(62–109)	(55–97)					
TC (mg/dL)	188	181	207	195	NS				
	(144–223)	(161–211)	(163–222)	(162–233)					
TG (mg/dL)	133	164	156	172	NS				
	(89–198)	(120–234)	(132–280)	(117–222)					
LDL-c (mg/dL)	110	99	96	128	NS	NS	0.036	0.002	NS
	(79–133)	(76–123)	(85–147)	(94–154)					
HDL-c (mg/dL)	45 (39–61)	45 (34–56)	41 (33–52)	47 (41–54)	NS				
Female	52 (43–64)	49 (42–60)	40 (31–57)	48 (42–54)					
Male	39 (34–52)	39 (30–51)	41 (33–55)	46 (40–53)					
Creatinine clearance (mL/min/24 h)	77 (60–95)	91 (72–140)	102 (62–126)	92 (77–112)	0.035	NS	NS	0.003	NS
Creatinine (mg/dL)	0.76 (0.64–0.96)	0.91 (0.73–1.26)	0.70 (0.64–0.95)	0.75 (0.64–0.93)	0.033	NS	NS	0.007	NS
C-Peptide (ng/mL)	0.2 (0.01–0.85)	2.4 (1.3–3.6)	1.14 (0.80–1.83)	1.87 (1.27–2.48)	<0.001	0.028	<0.001	0.019	NS
C-peptide after hyperglycemic exclusion	0.2 (0.01–0.95)	2.69 (1.3–3.6)	1.39 (0.77–2.0)	1.72 (0.80–2.30)	<0.001	0.036	<0.001	0.010	NS
Anti-GAD+ (n = 161)	55% (21/38)	4% (2/49)	0% (0/13)	23% (14/61)	0.001	NS	0.037	0.050	NS
Anti-IA2 + (n = 161)	42% (16/38)	0% (0/49)	0% (0/13)	62% (38/61)	0.007	NS	<0.001	<0.001	<0.001

T1D: type 1 diabetes; T2D: type 2 diabetes; LADA: Latent Autoimmune Diabetes in Adults; MODY: Maturity Onset Diabetes of the Young; NS: not significant; HbA1c: glycosylated hemoglobin A1c; TC: total cholesterol; TG: triglycerides; LDL-c: low density lipoprotein cholesterol; HDL-c: high density lipoprotein cholesterol; anti-GAD: glutamic acid descarboxylase antibodies; anti-IA2: tyrosine phosphatase antibodies. * Two patients with MODY were reclassified as T1D due to the positivity of anti-GAD. The results of quantitative variables are given in the median with an interquartile range. ** There were no statistical differences among patients with T1D vs. MODY, except for glucose concentration (*p* = 0.028) and C-peptide (*p* < 0.001).

**Table 3:** Use of diabetes medication by all subjects and those based on diabetes type.

Medication	T1D (n = 38)	T2D (n = 49)	MODY (n = 13)	LADA (n = 61)	*p * (T2D vs. T1D)	*p * (T2D vs. MODY)	*p * (T1D vs. LADA)	*p* (T2D vs. LADA)
Rapid-acting insulin (Humalog or lispro)	29	9	3	12	<0.001	NS	<0.001	NS
	(76%)	(18%)	(23%)	(20%)				
Intermediate-acting insulin (NPH)	14	24	4	25	0.001	NS	NS	NS
	(37%)	(49%)	(31%)	(41%)				
Long-acting insulin (glargine)	21	5	4	10	0.001	NS	<0.001	NS
	(55%)	(10%)	(31%)	(16%)				
Metformin	5 (13%)	27 (45%)	7 (54%)	42 (69%)	0.001	NS	<0.001	NS
Sulphonylurea	0 (0%)	4 (8%)	10 (77%)	17 (28%)	NS	<0.001	<0.001	0.006
Use of insulin	100%	29 (59%)	9 (69%)	33 (54%)	<0.001	<0.001	<0.001	NS
Insulin doses (U/kg)	0.80	0.50	0.61	0.12	<0.001	NS	<0.001	<0.001
	(0.60–1.0)	(0.31–0.67)	(0.39–0.65)	(0.04–0.51)				
Total insulin dose (U/day)	46.5	35	34	31	0.002	NS	<0.001	NS
	(38–70)	(25–48.5)	(27–55)	(18–42)				

X2 or Mann-Whitney U, according to the type of variable. *p* < 0.05 was considered significant. T1D: type 1 diabetes; T2D: type 2 diabetes.

**Table 4:** C-peptide correlation with anthropometric and biochemical parameters.

	Total Study Population		Type 1 Diabetes		Type 2 Diabetes		MODY		LADA	
	rho	*p*	rho	*p*	rho	*p*	rho	*p*	rho	*p*
Fasting C-peptide vs. weight	0.452	<0.001	0.524	0.012	0.499	0.001	0.446	NS	0.276	0.039
vs. BMI	0.390	<0.001	0.454	0.034	0.380	0.017	0.410	NS	0.317	0.019
vs. waist circumference	0.491	<0.001	0.430	NS	0.506	0.001	0.237	NS	0.212	NS
vs. WHtR	0.397	<0.001	0.369	NS	0.332	0.039	0.213	NS	0.142	NS
vs. TAG	0.408	<0.001	0.272	NS	0.507	0.001	0.005	NS	0.372	0.006
vs. HDL-c	−0.295	0.001	−0.403	NS	−0.326	0.049	−0.339	NS	−0.299	0.028

MODY: Maturity-Onset Diabetes of the Young: NS: not significant; BMI: body mass index; WHtR: waist-to-height ratio; HbA1c: glycosylated hemoglobin A1c; TC: total cholesterol; TG: triglycerides; HDL-c: high-density lipoprotein cholesterol.

**Table 5:** Multiple regression analysis for C-peptide concentrations.

Variable	Total Group		Type 2 Diabetes		Type 1 Diabetes		LADA	
	ß * (IC95%)	*p*	ß * (IC95%)	*p*	ß * (IC95%)	*p*	ß * (IC95%)	*p*
WC	0.468 (0.038–0.074)	<0.001	0.496 (0.033–0.107)	<0.001	NS		NS	
Insulin use	−0.280 (−1.351–−0.401)	<0.001	−0.288 (−2.093–−0.110)	0.030	NS		NS	
Sex	0.217 (0.207–1.178)	0.006	0.307 (0.183–2.211)	0.022	NS		NS	
BMI	NA		NS		0.482 (0.012–0.235)	0.031	NS	
HbA1c	NS		NS		NS		−0.280 (−0.227–−0.002)	0.047

WC: waist circumference; BMI: body mass index; HbA1c: glycosylated hemoglobin A1c. * Adjusted by sex, age, waist circumference, BMI, insulin use, HbA1c, fasting glucose, metformin, and sulphonylurea use.

## Data Availability

The data analyzed in this study are available from the corresponding author on reasonable request.
